# Long-term outcomes and evolving trends in thoracic aortic infections: A 25-year, single-center study in Japan

**DOI:** 10.1016/j.xjon.2025.10.004

**Published:** 2025-10-21

**Authors:** Katsuhiro Yamanaka, Kenji Okada, Daiki Kato, Hironaga Shiraki, Ryo Kawabata, Shunya Chomei, Taishi Inoue, Shota Hasegawa, Soichiro Henmi, Hiroaki Takahashi, Yutaka Okita

**Affiliations:** a Division of Cardiovascular Surgery, The Department of Surgery, Kobe University Graduate School of Medicine, Kobe, Japan; b Division of Cardiovascular Surgery, Cardio-Aortic Center, Takatsuki General Hospital, Osaka, Japan

**Keywords:** aortic surgery, stent graft, graft infection, aortoesophageal fistula, aortobronchial fistula

## Abstract

**Objectives:**

Thoracic aortic infection (TAI), including infections of the native aorta, prosthetic graft infections, aortoesophageal fistula, and aortobronchial fistula, remains among the most challenging and fatal diseases. This study aimed to review our 25-year experience with surgical management of TAI.

**Methods:**

This retrospective study included 106 participants with TAI from January 2000 to September 2024. The primary end point was hospital mortality. Secondary end points included 30-day mortality, trends in TAI management, overall survival, freedom from infection-related death, and freedom from infection-related events. Subgroup analyses were also conducted. The current surgical strategy has been in use since 2008.

**Results:**

Among 106 participants, 33 (31.1%) had aortoesophageal fistula, 7 (6.6%) had aortobronchial fistula, and 66 (62.2%) had TAI without fistula. Eighty-four participants underwent surgery after 2008. In situ replacement was performed in 85 (80.1%), thoracic endovascular aortic repair in 15 (14.1%), and extra-anatomical bypass in 6 (6%). The 30-day mortality rate was 3.7% (n = 4), and hospital mortality was 16.0% (n = 17). At 10 years, overall survival was 55.4% ± 5.9%, freedom from infection-related death was 78.7% ± 4.3%, and freedom from infection-related events was 76.1% ± 4.4%. Participants who underwent surgery after 2008 had significantly better outcomes than those treated before 2008.

**Conclusions:**

Despite the continued high hospital mortality associated with surgical treatment of TAI, the strategy implemented since 2008 has resulted in improved outcomes. The long-term outcomes were acceptable.

## Graphical Abstract

**Figure:**
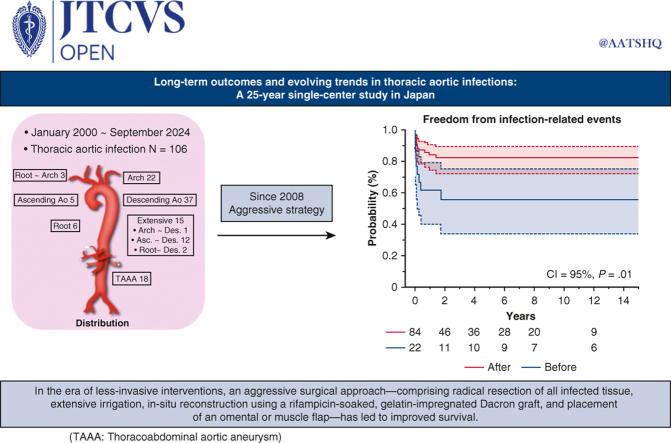

**Figure:**
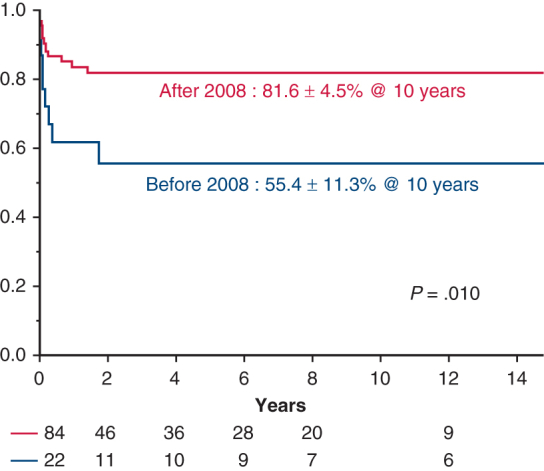
Freedom from infection-related events in the post-2008 group versus the pre-2008 group. 95% CI.

**Central Message**

TAI remains a formidable clinical challenge. Although hospital mortality remains high, the surgical strategy implemented since 2008 has yielded improved outcomes.

**Perspective**

Managing TAI is particularly challenging owing to patients’ critical condition. In the era of less-invasive interventions, an aggressive surgical approach—comprising resection of all infected tissue, extensive irrigation, in situ reconstruction using a rifampicin-soaked, gelatin-impregnated polyethylene terephthalate graft, and placement of an omental or muscle flap—has led to improved survival.

Thoracic aortic infection (TAI) is a rare but highly complex and life-threatening condition. Its high mortality is largely attributed to patients’ underlying comorbidities and clinical conditions. TAI encompasses a spectrum of pathological entities, including native aortic infection (NAI), prosthetic graft infection (PGI), aortoesophageal fistula (AEF), and aortobronchial fistula (ABF). Consequently, the management of patients with TAI is challenging and necessitates a multidisciplinary approach. Standard treatment includes appropriate antibiotic therapy, debridement of infected tissue, and graft replacement. However, endovascular repair has also been reported as effective.^1^ Owing to the limited sample sizes in previous single-center retrospective studies, no consensus has been established regarding the optimal surgical strategy. This study aimed to review 25 years of surgical experience with TAI at our institution.

## Methods

### Diagnosis and Study Design

This retrospective, single-center study was approved by the Institutional Review Board of Kobe University School of Medicine (#B220178; March 13, 2025), and the requirement for informed consent was waived.

A total of 106 patients with TAI were enrolled from January 2000 to September 2024. A multidisciplinary team—including infectious disease physicians, cardiovascular surgeons, plastic surgeons and radiologists—reviewed each case to determine treatment strategies. Diagnosis was based on clinical course, positive blood cultures, and enhanced computed tomography (CT), which typically showed irregular aneurysmal morphology, intramural gas, or contrast enhancement within low-density regions. Additional diagnostic tools included transesophageal echocardiography and 18-fluorodeoxyglucose (^18^F-FDG) positron-emission tomography (PET)/CT.^2^^,^^3^ Final diagnoses were confirmed using microbiological culture and histopathological analysis of intraoperative specimens in patients who underwent graft replacement.

The primary outcome was in-hospital mortality. Secondary outcomes included 30-day mortality, temporal trends in TAI, and long-term outcomes such as overall survival, freedom from infection-related death, and freedom from infection-related events. Infection-related events were defined as infection-related death or recurrent infection. Subgroup analyses were performed based on the presence of fistulas (AEF group, ABF group, or no fistula group), aortic reconstruction method (in situ replacement group, extra-anatomical bypass [EAB] group, or thoracic endovascular aortic repair [TEVAR] group), and infected tissue type (NAI or PGI). Long-term outcomes were also compared before and after 2008, when the current surgical strategy was implemented.

### Medical Treatment

Antibiotic therapy was administered both preoperatively and postoperatively, guided by culture results and drug sensitivity profiles. Regimens were selected based on the Sanford Guide to Antimicrobial Therapy (1999 to present) and American Heart Association guidelines.^4^ Antibiotics are determined based on the culture results and administered for 6 weeks. When cultures are negative, empiric therapy was administered. Thereafter, lifelong oral antibiotics are given in patients with residual prosthetic grafts or stent grafts, or at the discretion of the surgeon.

### Surgical Treatment

Surgical management involved debridement of infected tissues, extensive saline irrigation, and aortic reconstruction using in situ graft replacement, EAB, or TEVAR. Since 2008, our surgical protocol has included radical resection of all infected tissue, saline irrigation, and in situ replacement using a rifampicin-soaked, gelatin-impregnated polyethylene terephthalate graft, followed by omentopexy or a pedicled latissimus dorsi musculocutaneous flap (Video 1). A rifampicin-soaked, gelatin-impregnated polyethylene terephthalate graft was soaked in 0.4% rifampin solution for about 15 minutes before implantation. Silk sutures and felt strip were also soaked in rifampin. Before 2008, only abscess debridement was performed. Post-2008, radical resection encompassed abscesses, necrotic tissues, and the aortic wall. The surgical approach varied by infection site: midline incisions for infection from the aortic root to arch; the anterolateral thoracotomy with partial sternotomy for extensive arch involvement; and a left thoracotomy via the fourth or fifth intercostal space with rib-cross technique for descending or thoracoabdominal aortic infections.^5^ After cardiopulmonary bypass was initiated, complete resection was performed before aortic reconstruction. For patients with AEF, the esophagus was simultaneously resected.^5^ For those with ABF, pulmonary resection was completed before initiating cardiopulmonary bypass to prevent hemorrhage. Since 2008, in situ graft replacement has been the preferred method for aortic reconstruction. TEVAR was selectively performed in cases with localized infection, immunocompromised status (eg, advanced cancer), or as a bridge to open repair (bridge TEVAR), which was distinctly categorized in this study. Omentopexy was conducted concurrently with aortic reconstruction. When an omental flap was unavailable, a pedicled latissimus dorsi musculocutaneous flap was applied several days later. In patients with PGI and either multiple stent grafts or extensive aortic replacement, the resection area was sometimes guided by preoperative ^18^F-FDG PET/CT or intraoperative Gram staining of resected specimens. Furthermore, in patients with AEF, esophageal reconstruction is usually scheduled 2 or 3 months after discharge, following a period of rehabilitation in daily life.

### Statistical Analysis

Statistical analyses were conducted using JMP for Macintosh, version 18.1.1 (SAS Institute Inc). Continuous variables were presented as mean ± SE. Kaplan-Meier analysis was used to assess overall survival, freedom from infection-related death, and freedom from infection-related events.

## Results

### Patients’ Characteristics and Trends of TAI

**Figure 1:**
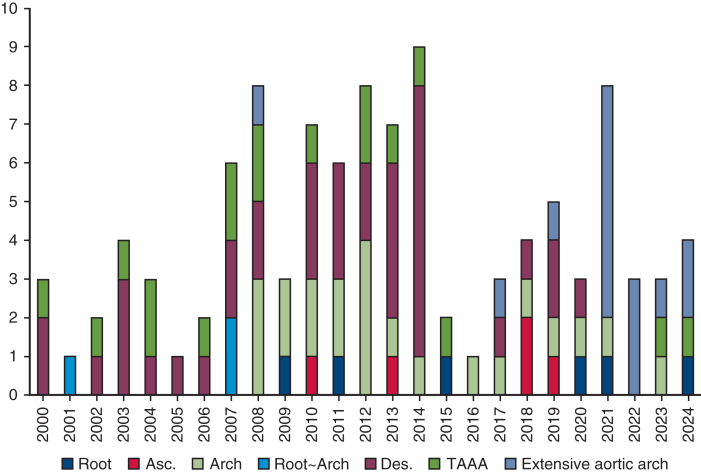
Annual number of procedures performed per patient. Patients were color-coded into 7 groups based on infection location. *Asc*, Ascending; *TAAA*, thoracoabdominal aortic aorta.

**Figure 2:**
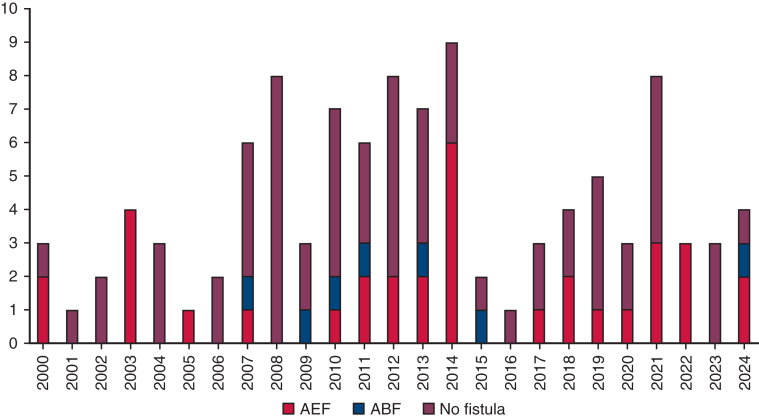
Trends in patients with aortoesophageal fistula (AEF), aortobroncheal fistula (ABF), or no fistula (2000-2024).

**Figure 3:**
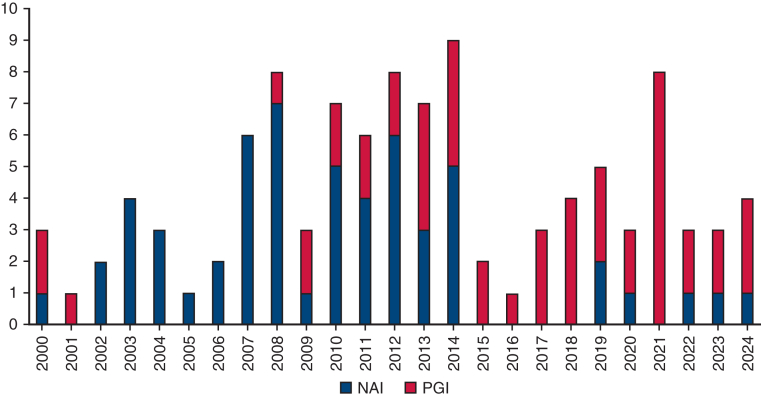
Trends in patients with prosthetic graft infection (PGI) or infection of native aorta (NAI) (2000-2024).

Between January 2000 and September 2024, 106 patients underwent surgery for TAI. Of these, 22 underwent surgery before 2008, and 88 after 2008. The mean age was 67.7 ± 12.6 years. Seventy-six patients (71.6%) were men. The anatomical distribution of TAI was as follows: aortic root in 6 patients (5.6%), ascending aorta in 5 (4.7%), aortic root to arch in 3 (2.8%), aortic arch in 22 (20.7%), descending aorta in 37 (34.9%), extensive aortic arch in 15 (14.1%), and thoracoabdominal aorta in 18 (16.9%). Figure 1 illustrates the annual number of patients, color-coded by infection location. AEF was present in 33 patients (31.1%), ABF in 7 (6.6%), and no fistula in 66 (62.2%). Among the 33 patients with AEF, 14 had primary AEF—owing to advanced esophageal cancer (n = 7), fish bone (n = 1), or aortic aneurysm (n = 6)—and 19 had secondary AEF. Among the 7 patients with ABF, three had secondary ABF, and 4 had primary ABF. No malignancy was observed in the ABF group. Figure 2 shows the trend in the presence or absence of fistula (AEF, ABF, or none) from 2000 to 2024. Fifty patients (45.5%) had PGI, whereas 56 (54.4%) had NAI. Figure 3 presents trends in PGI and NAI over the study period. Thirty-seven patients (34.9%) had comorbidities, including infectious disease (n = 10), malignant disease (n = 8), diabetes mellitus (n = 6), steroid use (n = 5), end-stage kidney disease requiring hemodialysis (n = 5), and liver failure (n = 3). Preoperative blood cultures were positive in 47 patients (45%). The most common pathogens were methicillin-resistant *Staphylococcus aureus* (MRSA) (n = 13) and *Streptococcus* spp (n = 7) (Table 1).

**Table 1:** Microbiological culture results

Culture	Result
Unknown	59 (55)
MRSA	13 (12)
*Streptococcus*	7 (6)
MSSA	4 (4)
*Pseudomonas*	4 (4)
*Escherichia coli*	3 (3)
*Candida*	3 (3)
*Salmonella*	3 (3)
*Enterococcus*	2 (2)
*Parvimonas micra*	2 (2)
Others	7 (6)

Values are presented as n (%). *MRSA*, Methicillin-resistant *Staphylococcus aureus*; *MSSA*, methicillin-sensitive *Staphylococcus aureus*.

### Operative Details

In situ replacement was performed in 85 patients (80.1%), including 5 who underwent bridge TEVAR. Six patients (5.6%) underwent EAB, and 15 (14.1%) underwent TEVAR. Among these 15 patients, TEVAR was the primary procedure in 4, and the causes for the remaining 11 were esophageal cancer (n = 6), liver cirrhosis (n = 1), high-dose steroid use (n = 1), dementia (n = 1), nonagenarian (n = 1), and fish bone (n = 1). Omentopexy was performed in 57 patients (53.6%), and 8 (7.5%) underwent reconstruction with a latissimus dorsi musculocutaneous flap.

### Early Outcomes

The 30-day mortality rate was 3.7% (n = 4). One patient without fistula and NAI died of residual aneurysm rupture 10 days postoperatively. The remaining 3 patients—1 without fistula and NAI, 1 with AEF and PGI, and 1 with ABF and PGI—died from multiple organ failure. Hospital mortality was 16.0% (n = 17): 18.1% for AEF, 28.5% for ABF, and 13.6% for no fistula. Mortality rates for NAI and PGI were both 16.0%. By procedure, mortality was 14.2% for in situ replacement, 33.3% for EAB, and 12.5% for TEVAR. Excluding 30-day mortality, among 13 hospital deaths, the causes were as follows: sepsis in 5, AEF in 2, pneumonia in 2, anastomotic bleeding in 1, aneurysm rupture in 1, pancreatitis in 1, and mediastinitis in 1.

### Late Outcomes

**Figure 4:**
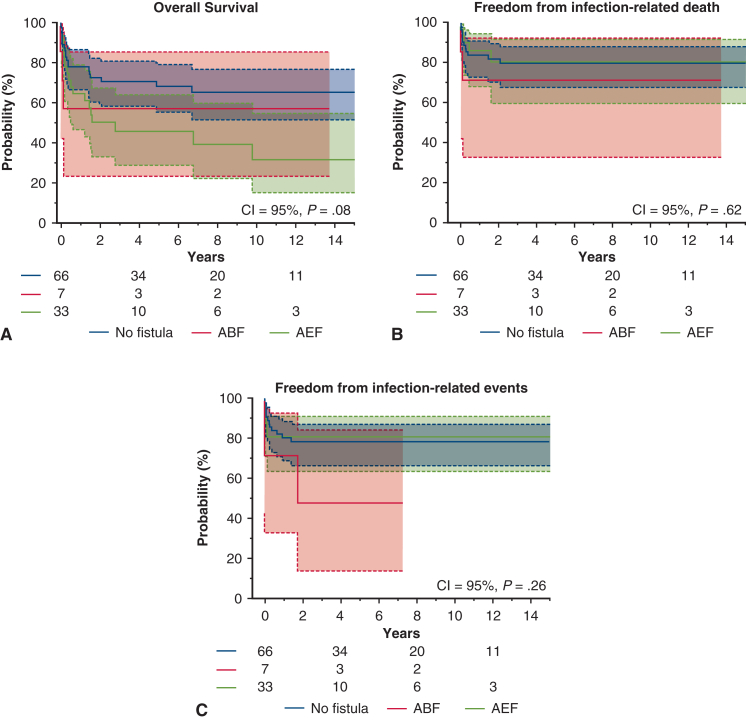
Kaplan-Meier curves for the aortoesophageal fistula (AEF) group versus the aortobronchial fistula (ABF) group versus the no fistula group. 95% CI. A, Overall survival. B, Freedom from infection-related death. C, Freedom from infection-related events.

**Figure 5:**
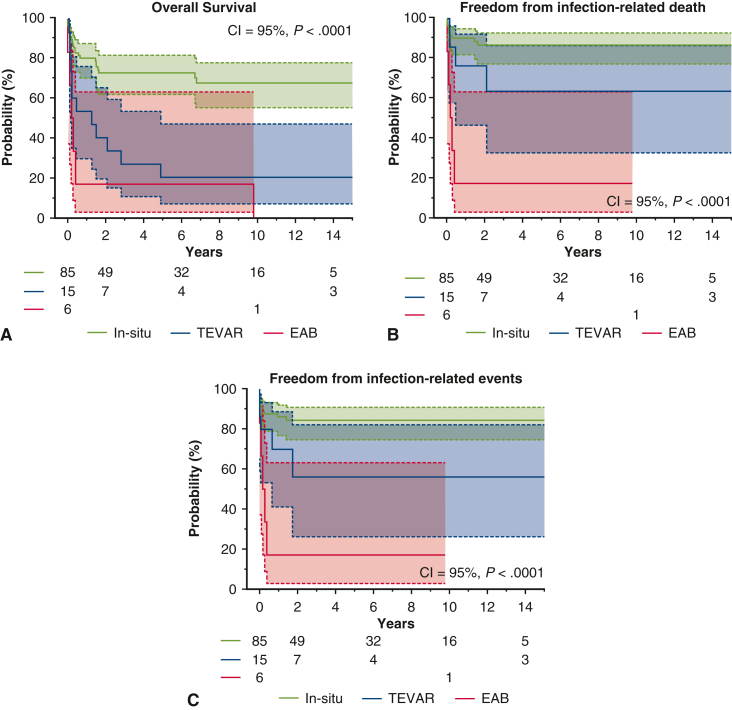
Kaplan-Meier curves for the in situ replacement group versus the thoracic endovascular aortic repair (TEVAR) group versus the extra-anatomical bypass (EAB) group. 95% CI. A, Overall survival. B, Freedom from infection-related death. C, Freedom from infection-related events.

**Figure 6:**
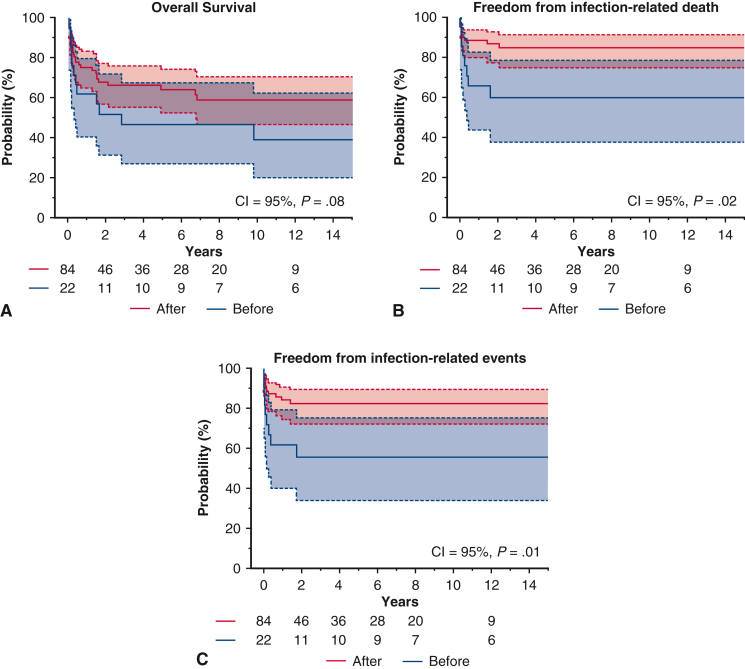
Kaplan-Meier curves for the pre-2008 group versus the post-2008 group. 95% CI. A, overall survival. B, Freedom from infection-related death. C, Freedom from infection-related events.

**Figure E1:**
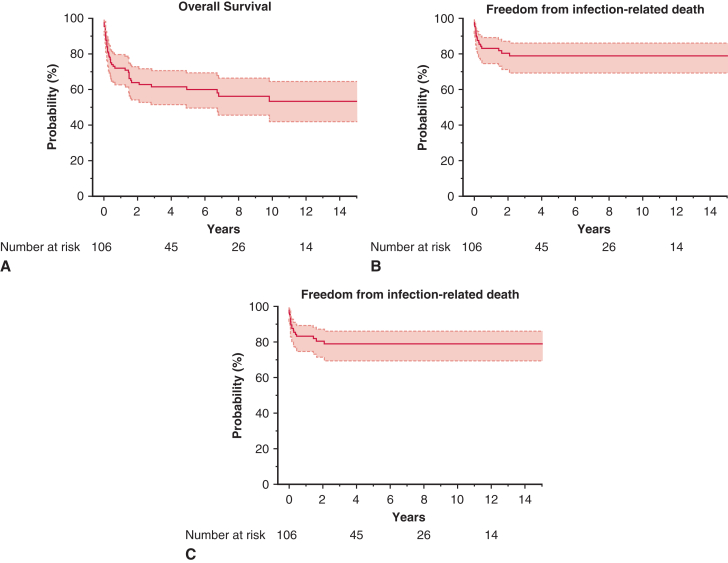
Kaplan-Meier curve for all participants. 95% CI. A, Overall survival. B, Freedom from infection-related death. C, Freedom from infection-related events.

**Figure E2:**
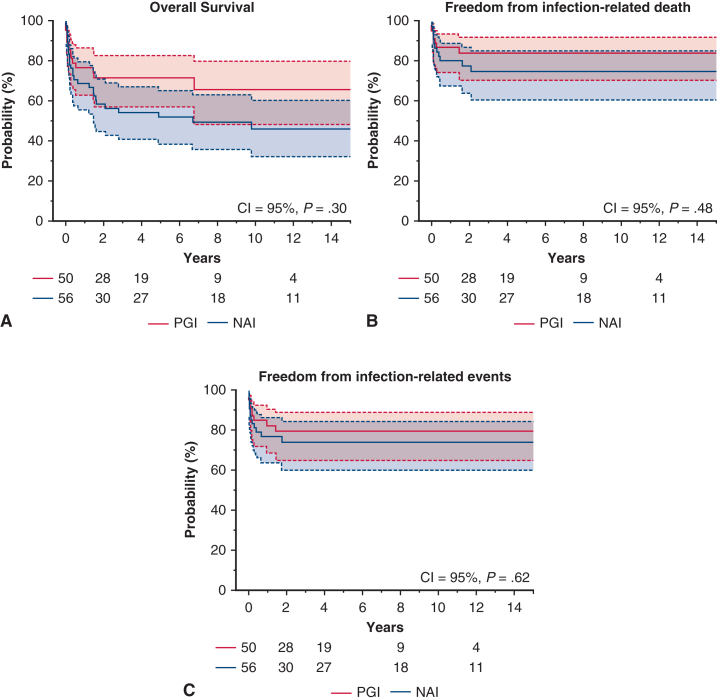
Kaplan-Meier curves for the prosthetic graft infection (PGI) group versus the infection of native aorta (NAI) group. 95% CI. A, Overall survival. B, Freedom from infection-related death. C, Freedom from infection-related events.

At 10 years, overall survival was 55.4% ± 5.9%, freedom from infection-related death was 78.7% ± 4.3%, and freedom from infection-related events was 76.1% ± 4.4% (Figures E1, *A*-*C*). Among the AEF, ABF, and no fistula groups, AEF had the lowest overall survival at 10 years (AEF: 31.2% ± 10.6%, ABF: 57.1% ± 18.7%, no fistula: 65.3% ± 6.5%), although differences were not statistically significant (*P* = .08) (Figure 4, *A*). No significant differences were observed among these groups in infection-related death or event-free survival (Figure 4, *B* and *C*). One patient in the ABF group experienced hemoptysis 1.7 years postoperatively. Among procedural groups, the 10-year overall survival was 67.6% ± 5.9% for in situ replacement, 18.7% ± 9.7% for TEVAR, and 0.00% ± 0.00% for EAB (*P* < .0001) (Figure 5, *A*). The in situ group had the highest freedom from infection-related death (86.8% ± 3.9%) and events (84.6% ± 4.1%) (Figure 5, *B* and *C*). In contrast, the EAB group had the lowest freedom from infection-related death (16.6% ± 15.2%) and events (16.6% ± 15.2%) (*P* < .0001) (Figure 5, *B* and *C*). When comparing PGI and NAI, no significant differences were found in overall survival, infection-related mortality, or event-free survival (Figure E2, *A-C*). When comparing surgeries performed before and after 2008, patients treated after 2008 had a 10-year overall survival of 61.1% ± 6.3%, compared with 38.7% ± 11.6% for those treated earlier (*P* = .08) (Figure 6, *A*). Freedom from infection-related death (84.0% ± 4.3% vs 59.7% ± 11.1%; *P* = .02) and events (81.6% ± 4.5% vs 55.4% ± 11.3%; *P* = .01) was significantly improved in patients treated after 2008 (Figure 6, *B* and *C*).

## Discussion

In this study, we reviewed our 25-year experience with the surgical management of TAI. A recent trend we observed was an increase in cases involving extensive aortic arch pathology. With the expanded use of TEVAR and the frozen elephant trunk technique for aortic arch repair, complications related to stent grafts, including infections, have become more common. Infection-related complications that required open conversion typically involved complex aortic arch pathology. In such cases, we prefer the anterolateral thoracotomy with partial sternotomy approach owing to its superior exposure and ability to protect multiple organs.^6^ Regarding aortic pathology trends, we consistently gained experience treating AEF across all years. The number of patients with NAI has decreased markedly, whereas the number of patients with PGI has increased significantly.

Organisms commonly responsible for aortic infection include gram-positive cocci (mainly *Staphylococcus*) and gram-negative bacilli (mainly *Salmonella*).^7^ According to a Mayo Clinic report,^8^ *Staphylococcus* accounted for 30%, *Streptococcus* 20%, *Salmonella* 20%, and *Escherichia coli* 15% of cases. In contrast, a report from National Taiwan University found that *Salmonella* was responsible for 76%, whereas gram-positive cocci accounted for approximately 10%.^9^ In this study, MRSA was the most commonly identified organism, found in 13 patients (12%). Furthermore, positive blood cultures were present in only 45% of patients. In other words, it should be noted that more than half (55%) of the patients had negative blood culture results, likely owing to prior prolonged antibiotic therapy. This problem may contribute to the difficulty in managing TAI. Therefore, the appropriate use of antibiotics is of critical importance. If cultures are negative, empiric therapy should be administered. After initiating empiric therapy preoperatively, we ensure reassessment with repeat blood cultures every 48 to 72 hours. We avoid unnecessarily prolonged broad-spectrum antibiotic therapy. Postoperatively, we continue empiric therapy for 6 weeks, and the subsequent use of lifelong oral antibiotics is considered on a case-by-case basis. Regarding lifelong oral antibiotics, even when enhanced CT or ^18^F-FDG-PET/CT shows no evidence of infection, lifelong oral antibiotics were basically continued, primarily to prevent reinfection.

Although the 30-day mortality rate was 3.7%, hospital mortality was higher at 16.0%, consistent with findings from other studies.^1^^,^^10^, ^11^, ^12^ Among subgroups, hospital mortality was especially high (50.0%) in patients with EAB. This procedure was likely chosen owing to the severity of their condition. All 6 patients who underwent EAB had surgery before 2008. Two of them died from stump rupture, a serious and clinically significant complication of the EAB procedure.^13^^,^^14^ A review article on EAB reported an incidence of stump rupture ranging from 3% to 50% and that the associated mortality was approximately 75%; however, more recent series showed lower rates.^15^ When EAB is unavoidable, thorough debridement, low suture tension, and anastomotic coverage with healthy tissue are of critical importance.^14^ Sarac and colleagues^16^ recommended the use of a fascia-peritoneum patch as a pledget to prevent stump rupture. To prevent stump rupture, we believe radical resection of infected tissue followed by in situ aortic reconstruction is essential.Some studies have reported similar survival rates between in situ reconstruction and EAB in patients with abdominal aortic infection, suggesting an individualized surgical approach.^14^^,^^17^ However, the use of EAB has declined owing to improved antimicrobial therapy and its low long-term patency rate.^17^^,^^18^ Although the TEVAR group (n = 15) had the lowest hospital mortality (6.6% [n = 1] sepsis), overall survival was poor owing to comorbidities. Additionally, both freedom from infection-related death and freedom from infection-related events were low in this group. Of 15 patients, 6 developed recurrent infections or infection-related complications. Recent meta-analyses have shown higher rates of infection-related complications after endovascular repair than open repair, although endovascular repair is associated with better short-term outcomes.^19^^,^^20^ Both a Japanese multicenter retrospective cohort study on AEF and the 1 on infected abdominal aortic aneurysms reported that open repair should be the first-line treatment for patients with abscesses adjacent to the aneurysm.^21^ Therefore, TEVAR should be reserved for high-risk patients unsuitable for open repair or used as a bridge to open repair.

Our current surgical strategy for TAI comprises 4 key components: radical resection of all infected tissues, abundant irrigation, in situ aortic reconstruction using rifampicin-soaked, gelatin-impregnated polyethylene terephthalate grafts, and installation of an omental or muscle flap.^22^ Each component is critical for therapeutic success, particularly part 1. Before 2008, only abscess debridement was performed, whereas after 2008, radical resection included not only abscesses and necrotic tissue but also the aortic wall including the surrounding tissues. In patients with AEF or ABF, esophagectomy or lung resection was not performed before 2008. This change has contributed to improved outcomes.^11^ For reconstruction, we use rifampicin-soaked, gelatin-impregnated polyethylene terephthalate grafts. Frankel and colleagues^11^ noted that prosthetic grafts are practical and effective in reducing recurrent infection. These grafts are suitable in most TAI cases.^23^^,^^24^ Although biofilm formation on prosthetic grafts reduces antimicrobial efficacy, rifampicin can disrupt *Staphylococcus* biofilms.^25^ Sasajima and colleagues^26^ found that these grafts maintain antibacterial activity for 2 weeks and are effective against *Staphylococcus epidermidis*, but not against MRSA or *E coli*. Biological conduits such as autologous superficial femoral vein,^16^^,^^27^ cryopreserved arterial allograft,^22^^,^^28^ and xenopericardial tubes^29^^,^^30^ have also been used. However, each has limitations. Femoral vein grafts have diameter mismatches and are limited in length, requiring harvesting time.^11^^,^^27^ Homograft is the ideal conduit in the setting of TAI. However, the use of homografts is limited in some countries due to the lack of tissue banking systems. The homografts may cause early and late degeneration owing to primitive preservation methods.^24^^,^^28^ Xenopericardial tubes lack sufficient long-term comparative data on calcification and durability.^31^^,^^32^ Considering these reasons, we most commonly use the rifampicin-soaked polyethylene terephthalate graft. Regarding part 4, the omentum provides rich blood flow and lymphoid tissue, offering absorptive and immunologic advantages.^33^ Its amorphous structure allows it to fill cavities and shield grafts. When omental flaps were unavailable owing to prior laparotomies or gastrectomy, pedicled latissimus dorsi musculocutaneous flaps were used. Sandhu and colleagues^10^ showed that autologous tissue coverage improves outcomes in thoracic aortic graft infection. Omentopexy was performed concurrently with aortic reconstruction, whereas musculocutaneous flaps were installed in a staged approach owing to harvesting complexity.

Comparing outcomes between NAI and PGI groups, no significant differences were observed, contrary to expectations. We anticipated worse outcomes in the PGI group owing to pleural adhesions and poorer general condition. Additionally, these patients often had multiple stent grafts or extensive aortic replacements, making complete graft resection difficult. The ^18^F-FDG-PET/CT scan is valuable for detecting graft infection^3^ and helps determine resection extent.^34^ Our surgical strategy, including all 4 components and tailored resection, likely contributed to favorable outcomes.

Pathologic communication (AEF and ABF) between the thoracic aorta and esophagus or tracheobronchial tree is a severe condition, classified as primary or secondary. Primary causes include aortic aneurysm, malignancy, and foreign body ingestion, whereas secondary causes involve interventions such as aortic replacement or TEVAR.^35^ Secondary cases have increased.^21^ The 5-year survival rate of 44.2% ± 9.2% was consistent with previous findings.^35^ In this study, 33 patients had AEF; 7 had advanced esophageal cancer and underwent TEVAR, whereas no malignancy was observed in the ABF group, accounting for the poorer long-term survival in the AEF group. Notably, freedom from infection-related death and infection-related events in the AEF group was comparable to the no-fistula group. One patient died of rupture 1.6 years postoperatively, likely owing to inadequate debridement before 2008, despite undergoing in situ reconstruction twice. No other long-term infections were identified. We perform simultaneous esophageal resection with infected tissues under cardiopulmonary bypass before in situ reconstruction. Additionally, bridge TEVAR is employed to stabilize hemodynamics.^5^ These strategies may explain the favorable long-term outcomes. One patient in the ABF group developed hemoptysis 1.7 years after TEVAR and required open conversion, after which no further infection occurred. This highlights the need for careful evaluation of TEVAR in patients with TAI.

### Limitations

This study has limitations. It is a retrospective, nonrandomized, single-center study with a small sample size. Group comparisons are limited by the small number of participants, particularly in the EAB, AEF, ABF, and pre-2008 subgroups. Additionally, histopathologic examinations were not conducted in patients treated with TEVAR alone.

## Conclusions

TAI continues to be associated with high in-hospital mortality. Our current surgical strategy—adopted after 2008—includes radical resection of all infected tissue, abundant irrigation, in situ reconstruction with a rifampicin-soaked, gelatin-impregnated polyethylene terephthalate graft, and installation of an omental or muscle flap. This approach has led to improved outcomes. Long-term results have been acceptable.

## Conflict of Interest Statement

The authors reported no conflicts of interest.

The *Journal* policy requires editors and reviewers to disclose conflicts of interest and to decline handling or reviewing manuscripts for which they may have a conflict of interest. The editors and reviewers of this article have no conflicts of interest.

## Supplementary Data

Video 1 The left side is caudal and the right side is cranial. This video demonstrates thoracoabdominal aortic replacement for descending aortic infection with an aortopulmonary fistula. Radical resection of all infected tissues was performed, and the aortic wall was resected en bloc with a portion of the left lower lobe. Aggressive debridement and further resection of infected aortic wall were carried out. The aorta was reconstructed with a rifampicin-soaked, gelatin-impregnated Dacron graft, followed by omentopexy. Video available at: https://www.jtcvs.org/article/S2666-2736(25)00369-9/fulltext.
