# Supplementary figures and images for: Long-term outcomes and evolving trends in thoracic aortic infections: A 25-year, single-center study in Japan
Source: JTCVS Open. 2025 Oct 21;28:1–12. doi: 10.1016/j.xjon.2025.10.004 (PMC12745102; doi:10.1016/j.xjon.2025.10.004)

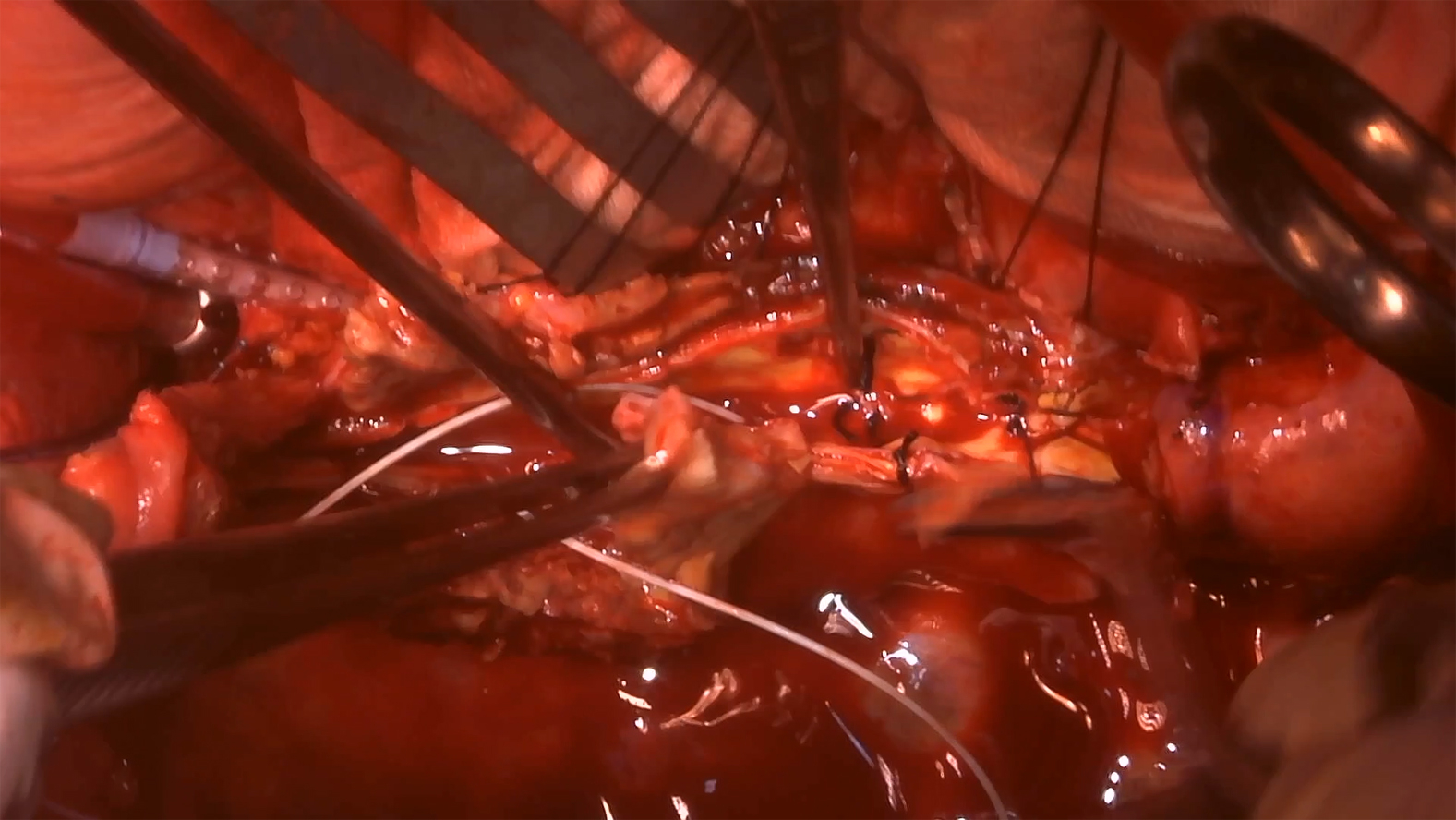

Supplement: Video 1 — The left side is caudal and the right side is cranial. This video demonstrates thoracoabdominal aortic replacement for descending aortic infection with an aortopulmonary fistula. Radical resection of all infected tissues was performed, and the aortic wall was resected en bloc with a portion of the left lower lobe. Aggressive debridement and further resection of infected aortic wall were carried out. The aorta was reconstructed with a rifampicin-soaked, gelatin-impregnated Dacron graft, followed by omentopexy. Video available at: https://www.jtcvs.org/article/S2666-2736(25)00369-9/fulltext. [file fx2.jpg]
